# The Rational Treatment of the Morphia Habit

**Published:** 1899-11

**Authors:** 


### THE RATIONAL TREATMENT OF THE MORPHIA HABIT.

The practice of employing Alcohol, Chloral, Ether, or other substitutes for Opium invariably results in grafting a new drug upon the old one. The employment of coco-wine, which has been highly recommended for use in cases of this sort, should be condemned in the strongest terms, for as is now thoroughly well known, these preparations are nothing more or less than a solution of Cocain in Alcohol.

The victim of the drug habit is usually a person of nervous temperament, and in many instances the drug habit is acquired as the result of a morbid condition of the nervous system which renders pain unendurable without the mitigating influence of some narcotic.—*Modern Medicine*, Aug., 1899.

